# Acknowledgment to Reviewers of *Antioxidants* in 2021

**DOI:** 10.3390/antiox11020326

**Published:** 2022-02-08

**Authors:** Antioxidants Editorial Office

**Affiliations:** MDPI AG, St. Alban-Anlage 66, 4052 Basel, Switzerland

Rigorous peer-reviews are the basis of high-quality academic publishing. Thanks to the great efforts of our reviewers, *Antioxidants* was able to maintain its standards for the high quality of its published papers. Thanks to the contribution of our reviewers, in 2021, the median time to first decision was 16 days and the median time to publication was 35 days. The editors would like to extend their gratitude and recognition to the following reviewers for their precious time and dedication, regardless of whether the papers they reviewed were finally published:
Aaronson, Philip I.Lawson, CharlotteAbate, GiuliaLazar, ZsofiaAbegg, DanielLázaro-Diéguez, FranciscoAbenavoli, LudovicoLazzarato, LorettaÁbrányi-Balogh, PéterLazzarino, GiacomoAcero, NuriaLeahu, AnaAckley, BrianLecewicz, MarekAcuña-Castroviejo, DaríoLech, KrzystofAdameova, AdrianaLechel, AndreAdamiano, AlessioLedgerwood, Elizabeth C.Adunyah, Samuel E.Lee, CheoljuAfonso, Andrea Luísa Fer-nandesLee, Der-YenAfrin, SadiaLee, Eun-WooAgnisola, ClaudioLee, Hong-JinAguilar-Zarate, PedroLee, Hwa-JinAhmad, FarazLee, HyeminAhmedova, AnifeLee, In-ChulAhn, Sung SooLee, I-TeAigotti, RiccardoLee, Jae WookAitken, R. JohnLee, Jetty Chung-YungAkbar, MohammedLee, Jin-YongAkita, SadanoriLee, Ji-YunAlaimo, AlessandroLee, JunsooAlbertini, MariaLee, Kwang YoulAlbini, AdrianaLee, KyungminAlcain, Francisco J.Lee, Li-AngAldasoro, MartinLee, Li-JenAldini, GiancarloLee, MinaAledo, CarlosLee, Myoung-SookAlessia, RosiLee, Sang KiAlexa, ErsiliaLee, Sang-HanAlexandre, AndreaLee, Soo YoungAleynova, Olga A.Lee, Sung KiAlhadidi, QasimLee, TaesupAliverti, AlessandroLee, Tzong-ShyuanAllais, FlorentLee, Yu-HsuanAlloza, IraideLegartová, SoňaAl-Maawi, SarahLegaz, IsabelAl-Maharik, NawafLegembre, PatrickAlmajano, María PilarLegrand, François-XavierAlmasi, ShekoufehLehotský, JánAlmeida, HenriqueLeinonen, Hanna M.Almeida, Maria JuditeLeitão, AnaAlonso-Torre, SaraLeite, Fábio R.M.Alov, PetkoLelario, FilomenaAlsalahi, RashadLemieux, HélèneAltintas, Mehmet MLenaz, GiorgioAlvarez, Ana I.Lendvai, GáborAlvarez-Rodriguez, Ma-nuelLentini, GiovanniAlvariño, RebecaLenzi, PaolaAlves, C. HenriqueLeo, Chen HueiAmarowicz, RyszardLeón, JosefaAmigo, LourdesLeondaritis, GeorgeAmoutzias, GrigorisLeon-González, Antonio J.Ampofo, EmmanuelLeontopoulos, StefanosAnas, Andrea Roxanne J.Lephart, Edwin D.Anastassova, NedaLeri, ManuelaAndjelkovic, MirjanaLescure, AlainAndo, HideyaLeslie, JackAndrade, IsabelLešnik, SamoAndrei, SandaLeszczyńska, JoannaAndrews, Melissa R.Lete, IñakiAndrzej, NowickiLetek, MichalAnesi, AndreaLever, JeremieAnestopoulos, IoannisLevy, EmileAngeletti, AndreaLewandowicz, GrażynaAngelini, CorradoLewandowska, HannaAngelino, DonatoLewandowska, KatarzynaAngelova, AngelinaLewinska, AnnaAngerhofer, AlexanderLeyva-Porras, César C.Anifandis, GeorgeLezza, Angela Maria Sere-naAnna, Chlebowska-ŚmigielL'homme, LaurentAnsari, RaisLi, Chia-JungAntoni, GunnarLi, Chien-FengAntonio Malfa, GiuseppeLi, Hsing-HuiAntonkiewicz, JacekLi, LinAntonopoulou, EfthimiaLi, LiyaAntonucci, SalvatoreLi, Ruei-NianAntunes, JoanaLi, WeiAntunes, Joana C.Li, YangAoyama, KojiLiagre, BertrandApolloni, SavinaLiao, ChangApostolidis, LeonidasLiao, Jyh-feiAprile, Francesco A.Lichtenberg, DovAprodu, IulianaLicini, CaterinaAraújo, MargaridaLightfoot, Adam P.Araújo, Maria EduardaLigor, MagdalenaAraujo, Maria E.M.Lim, Kyung-MinArboix, AdriàLim, Lee WeiArcaro, AlessiaLima, BeatrizArciszewski, MarcinLin, Chih-ChingArdalan, MaryamLin, Chih-LiArellano, Juan B.Lin, Chun-PingArfuso, FrancescaLin, Hui-HsuanArgenziano, MarianaLin, Jer-AnArguelles, SandroLin, RuweiArias, AndresLin, Shian-RenArmand, Anne-SophieLin, Wei-NingArmengol, José ÁngelLin, Yuh-JyhArmengol, RamónLinkies, AdaArnaud, ClaireLintig, Johannes VonArnhold, JürgenLio, DomenicoArnoux, PascalLiobikas, JuliusÅrøen, AsbjørnLionetto, Maria GiuliaArosio, PaoloLiou, Gunn‐GuangArroba, Ana ILiou, Je-WenArteaga, Jesús FernándezLipińska, BarbaraArtieda, Diana AnsorenaLiptrott, NeillArtuch, RafaelLiszka-Skoczylas, MartaArtunc, FerruhLittle, BertArvanitis, DemetriosLiu, Bing-LanAsano, KazuhitoLiu, Chia-ChiAssanelli, DeodatoLiu, Jun-HongAssari, ShervinLiu, Keng-KuAssi, MohamadLiu, Ming-CheAssy, NimerLiu, NingAsuero, Agustín G.Liu, Shing-HwaAtanassova, MariaLiwinienko, GrzegorzAthanasiou, Labrini V.Lizard, GérardAtlante, AnnaLlansola, MartaAullon, GabrielLlopis, JuanAvato, PinarosaLlorente, IreneAvdonin, Pavel V.Lo Schiavo, FiorellaÁvila, JulioLocatelli, ChiaraAvni, DoritLodovici, MauraAziz, Abdul RashidŁodyga-Chruścińska, ElżbietaAzuma, ChiekoLodygin, EvgenyAzzouz, AbdelmonaimLohan, SilkeBaby, André RolimLoi, MartinaBaczek-Kwinta, RenataLoizzo, Monica R.Badiale Furlong, ElianaLoizzo, Monica RosaBadino, PaolaLojk, JasnaBae, Chang-HyuLojkova, LeaBae, JiyoungLomakina, Anna V.Bae, Ok-NamLombardo, MauroBae, Woong JinLonardo, AmedeoBahnson, EdwardLončar, Mirela BausBaiocco, PaolaLong, ManhaiBajguz, AndrzejLópez Castellano, AliciaBajor, MalgorzataLopez Domenech, SandraBak, KathrineLópez Lluch, GuillermoBaker, MartinLópez Román, Francisco JavierBalažová, AndreaLópez, Lucía MéndezBalcerczak, EwaLópez-García, Juan CarlosBalcerczyk, AnetaLopez-Jornet, PiaBalciunaite, GabrieleLorenc-Koci, ElżbietaBaldanzi, GianlucaLorenz, KristinaBaldisserotto, AnnaLorenzo, Jose M.Bališ, PeterLortz, StephanBalla, JozsefLoßow, KristinaBallmann, ChristopherLourenço, MartaBalsera, MonicaLu, Chi-YuBaltazar, GracaLu, Wen-ChienBalzamino, Bijorn OmarLucero, DiegoBalzaretti, Claudia MariaLuchetti, FrancescaBandyopadhyay, DebasishLuciński, RobertBanfi, GiuseppeLuddi, AliceBang, John J.Ludwig-Słomczyńska, Ag-nieszka H.Bani, DanieleLukasiewicz, MarcinBaniene, RasaLukaski, HenryBano, ShaziaLukatkin, A. S.Banskota, Arjun H.Lukinac Čačić, JasminaBanti, Christina N.Lundegaard, Pia RengtvedBaptista, Filipa I.Lupi, Saturnino MarcoBaran, NataliaLutfy, KabirullahBarančík, MiroslavLymberopoulos, AristotelisBaranowska-Kuczko, Mar-taLyons, Daniel MarkBarbosa-Pereira, LetriciaM. Vorob'ev, MikhailBarbouti, AlexandraMa, KesenBárcena, José AntonioMa, Ming-chiehBarcena, Maria LuisaMa, SihuiBarczynski, BartlomiejMa, Wen-LungBareja, AkshayMaccallini, CristinaBar-Haim, ErezMaccari, FrancescaBarišić, LidijaMachado-Oliveira, GiselaBarnes, Michael E.Maciej, StrzemskiBaron, ByronMaciejczyk, MateuszBarragán, MontserratMackrill, JohnBarratt, GillianMadigan, MicheleBarreca, DavideMadrid, MarisaBarreto, MariaMadureira, Patrícia A.Barreto, Maria CarmoMaeng, Sung HoBarteková, MonikaMaestre Pérez, Salvador E.Bartolomeo, GiovanniMaestro, MiguelBartosz, GrzegorzMaffia, MicheleBasen, MirkoMagalhães, JoanaBasini, GiuseppinaMagder, Sheldon A.Baslam, MarouaneMagierowski, MarcinBassi, Anna MariaMahtouk, KarèneBassols, AnnaMaiese, AnielloBattistuzzi, GiannantonioMaietti, AnnalisaBaucum, Anthony J.Maioli, MargheritaBauer, IngeMaioli, SilviaBauer, JessicaMaita, MasashiBauer, MartinMaixent, Jean MichelBauer, ReinhardMajewska, MałgorzataBautista-Aguilera, Óscar MMajewski, MichałBauzá Thorbrügge, MarcoMakedou, KaliBavec, AljošaMakridis, AntoniosBaykov, SergeyMakuch, EdytaBazopoulou, DaphneMalaguti, MarcoBazwinsky-Wutschke, IvonneMalawska, BarbaraBeáta, LontayMalfa, GiuseppeBeberok, ArturMalik, Anna R.Beck, Michael W.Malińska, DominikaBecker, Katrin AnneMalle, ErnstBednarski, Patrick J.Maloberti, AlessandroBeharry, Kay D.Maltar-Strmečki, NadicaBehringer, ErikMalysh, Julia M.Beilby, MaryMambu, LengoBell, Aaron W.Manca, Maria LetiziaBellavite, PaoloMancardi, DanieleBello, Nicholas T.Mandal, AmritlalBelosludtseva, NataliaMandrich, LuigiBelousov, VsevolodMandrioli, RobertoBeltrán Sanahuja, AnaManetti, MirkoBeltran-Alacreu, HectorMáñez, SalvadorBelyaeva, Olga V.Manfra, MicheleBełżecki, GrzegorzMania, AnnaBencini, AndreaManini, PaolaBendich, AdrianneMannino, GiuseppeBenelli, RobertoManríquez-Torres, José De JesúsBenmoussa, AbderrahimMansueto, GelsominaBenohoud, MeryemMarabini, LauraBerardo, ClarissaMaragou, Niki C.Béraud-Dufour, SophieMaráková, KatarínaBercu, MioaraMaraver, FranciscoBeretta, Giovanni L.Marcal, HelderBerezin, AlexanderMarcocci, LuciaBergen, WernerMarco-Contelles, JoséBergqvist, AndersMarcolongo, PaolaBerhow, MarkMarculescu, RodrigBernad, AntonioMarengo, BarbaraBernardo, AntoniettaMargaritelis, NikosBernstein, Hans-GertMarginǎ, DenisaBerrino, EmanuelaMarí, MontserratBertero, LucaMaria Carmela, Bonaccorsi Di PattiBerthon, Annabel S.Mariani, PaoloBerti, FedericoMariastefania, AnticaBerto, SilviaMarin, LuminitaBevc, SebastjanMarini, CeciliaBhak, JongMarini, ElisabettaBhalla, ManmeetMarini, Herbert RyanBhatt, PriyankaMarini, MarinaBhattacharya, PratipMarino Gammazza, An-tonellaBhawal, Ujjal KumarMarino, GabrieleBhuiyan, AlauddinMariychuk, RuslanBi, LanrongMarkiewicz, RoksanaBiagi, MarcoMarongiu, Maria LauraBiagini, GiuseppeMarrelli, MassimoBiałek, MałgorzataMarsche, GuntherBianco, ArmandodorianoMarsili, StefaniaBianco, FrancescoMarsillach, JuditBielański, PawełMarsolais, FredericBijak, MichałMartella, GiuseppinaBikov, AndrásMartin Cordero, CarmenBilia, Anna RitaMartín, AlbertoBillon, CyrielleMartin, KeithBilska-Wilkosz, AnnaMartin, MariaBilski, JanMartín, María AntoniaBinkowska, IwonaMARTIN, MichèleBinninger, DavidMartinez, Francisco Edu-ardoBiondi, MarcoMartínez, LorenaBiringer, RogerMartínez, SidoniaBizoń, AnnaMartínez-Martínez, AlejandroBjörklund, SebastianMartinez-Pastor, FelipeBjornstedt, MikaelMartínez-Pinilla, EvaBlanchet, GuillaumeMartín-Gil, JesusBlangetti, MarcoMartinho, J. M G.Blank, RalfMartín-Montalvo, AlejandroBlanton, CynthiaMartino, Tartaglia Gianlu-caBlasi, FrancescaMartins, DianaBlasiak, JanuszMartins, FatimaBleilevens, ChristianMartins-Bessa, AnaBlenda, AnnaMartín-Vasallo, PabloBlengini, CeciliaMaruyama, KeiBloksgaard, MariaMarx, ChristianBoard, MaryMary, DidierBobis, OtiliaMasarone, DanieleBoccia, Antonella CaterinaMas-Bargues, CristinaBocianowski, JanMaserti, Bianca ElenaBodea, Liviu-GabrielMassai, LaraBody-Malapel, MathildeMassányi, PeterBoeldt, Derek SMassimino Cocuzza, Giuseppe ErosBoga, José AMastanjević, KristinaBogado Pascottini, OsvaldoMasternak, JoannaBogdanov, PatriciaMatei, IoanaBojić, MirzaMaterska, MałgorzataBölcskei, HedvigMatsubara, ShigekiBollong, MichaelMatsubara, TakumaBombik, ElżbietaMatsukawa, NoriyukiBonay, MarcelMatsuoka, KiwamuBonciu, ElenaMatteucci, ClaudiaBonior, JoannaMattison, Christopher P.Bonnet, CrystelMattsson, Mats-OlofBonomi, AliceMatusovsky, OlegBonomini, FrancescaMauricio, Maria DoloresBonsembiante, FedericoMaurizio, SABBATINIBontempo, PaolaMavrogonatou, EleniBordin, LucianaMazurek, HenrykBorges Dos Santos, Rui M.Mazzola, Eugene P.Borgonovo, GigliolaMazzola, Silvia MichelaBorras, ConsueloMazzucco, EleonoraBorrelli, EmmaMcKinstry, Karl KaiBoschi-Muller, SandrineMcStay, Gavin P.Boscia, FrancescaMeans, Robert T.Bosma, Piter JabikMechrez, GuyBotelho, Maria FilomenaMedina Juarez, Luis AngelBotez, ElisabetaMeinhardt, MatthiasBottari, SergeMekinić, Ivana GeneralićBouchal, JanMele, MirandaBouitbir, JamalMelendez-Martinez, Anto-nioBoulton, Michael E.Melkani, Girish C.Bourdon, EmmanuelMelo, AndréBourgeois, DenisMendiola León, Jose Anto-nioBousiges, OlivierMendonça, DinaBozic, JoskoMenegatti, EricaBrabet, PhilippeMenegazzi, MartaBraconi, DanielaMeneguzzo, FrancescoBrągoszewski, PiotrMenghini, LuigiBrainina, Khiena Z.Menon, Manoj B.Brandão, ElsaMerah, OthmaneBranowska, DanutaMerchán, MIguel ÁngelBratcher, Preston E.Merecz-Sadowska, AnnaBraun, FrankMerino, BeatrizBraun, RalfMertas, AnnaBrčić Karačonji, IrenaMertlíková-Kaiserová, HelenaBrecker, LotharMerwid-Ląd, AnnaBreitenbach, MichaelMesa, FranciscoBrestic, MarianMessina, AntoniettaBriedé, JaccoMestre-Francés, NadineBrigelius-Flohe, ReginaMetzinger, LaurentBrígido, ClarisseMézes, MiklósBroadhurst, C. LeighMiastkowska, MalgorzataBronikowski, MichałMicale, NicolaBros, MatthiasMicek, AgnieszkaBroskey, Nicholas T.Michael, AdrianBrouillet, EmmanuelMichel, PiotrBrown, DennisMichels, AlexBrown, PeterMichels, AlexanderBrownlee, Iain AMichels, Alexander J.Bruder Do Nascimento, ThiagoMichiels, CarineBrukman, NicolasMichna, AnetaBrunetti, CeciliaMicillo, RaffaellaBrunetti, LuigiMiękus, NataliaBrünnert, DanielaMiele, RossellaBruno, Giovanni L.Mierzejewska, JolantaBrycki, BogumilMieszczakowska-Frąc, MonikaBrzeziński, KrzysztofMieyal, John J.Bucciantini, MonicaMiguel, Maria Da Graça CostaBucolo, ClaudioMikhailov, OlegBucova, MariaMiklós, ZsuzsannaBūdienė, JurgaMikolajczak, PrzemyslawBudzisz, ElżbietaMilani, ChiaraBuentello-Volante, BeatrizMilano, SerenaBuffoli, BarbaraMilazzo, Maria FrancescaBuitrago-Molina, Laura ElisaMiller, IngridBulaev, AleksandrMiller, Neil RichardBulgariu, LauraMiloshev, GeorgeBunea, AndreaMimura, JunseiBungau, SimonaMin, HyeyoungBunik, VictoriaMin, KisukBurge, Kathryn Y.Min, Won LeeBusinaro, RitaMinafra, LuigiBusnelli, MarcoMinato, Ken-ichiroBusto, RebecaMinetti, GiampaoloButcher, AdrianMiniero, Daniela ValeriaButtari, BrigittaMinnelli, CristinaBuxadera-Palomero, JuditMinor, Radiah C.Buzanska, LeonoraMintāle, IvetaBystrom, JonasMinucci, SergioByun, SanguineMinutolo, AntonellaC Beitz, DonaldMiranda, CristobalCaballero-Garcia, BeatrizMiranda, KatrinaCacciola, FrancescoMircea, CorneliaCadamuro, MassimilianoMirete, SalvadorCai, GiampieroMirlekar, BhalchandraCai, HongmeiMiro, JordiCailliau, KatiaMisaki, KentaroCairrão, ElisaMiserocchi, GiuseppeCalado, Cecília R.C.Mishima, EikanCaldara, MarinaMishina, MasahiroCalderon-Montaño, Jose ManuelMiskulin, MajaCaleja, CristinaMita, GiovanniCălinoiu, Lavinia FlorinaMitidieri, EmmaCalín-Sánchez, ÁngelMitliagka, ParaskeviCalle, Mariana CeciliaMitov, MihailCalvert, JohnMitrofanova, AllaCalzia, EnricoMitsou, EvgeniaCambiaghi, MarcoMitsuaki, MoriyamaCampanella, AlessandroMiyamoto, IkuyaCampise, MariarosariaMiyata, JunCampo, Giuseppe Mau-rizioMiyauchi, SeijiCampos Rosa, Joaquín Ma-ríaMizukami, HirokiCampos, CatarinaMlejnek, PetrCampos, DeboraMleko, StanisławCanepari, MonicaMochida, KazuhikoCanini, AntonellaModica, MariaCanipari, RitaModun, DarkoCannizzaro, CarlaMogi, MasakiCano LaMadrid, MarinaMogielnicka-Brzozowska, MarzenaCano, AinaraMohamed Abdallah, Hos-samCansian, Rogério LuisMohanty, BijayalaxmiCantacorps, LidiaMohora, MariaCantamessa, SimoneMollace, VincenzoCapasso, DomenicaMollica, AdrianoCapasso, RaffaeleMonfrini, EdoardoCaporale, AndreaMonné, MagnusCapote-Puente, RaúlMonsalve, MariaCappai, Maria GraziaMontag, DirkCappelletti, GraziellaMontana, GiovannaCapperucci, AntonellaMontecucco, FabrizioCarata, ElisabettaMonteil, CarolineCardador-Martínez, Ana-bertaMontenegro, LuciaCardona, FernandoMontiel-Duarte, CristinaCardoso, ArmandoMontone, Carmela MariaCardullo, NunzioMontserrat-de La Paz, Ser-gioCarmen, MejíaMoore-Carrasco, RodrigoCarneiro, LionelMoosmann, BerndCarnevale Neto, FaustoMorales Suárez-Varela, María M.Carnevale, RobertoMorbidelli, LuciaCarotti, SimoneMorciano, GiampaoloCarrascal, LiviaMordvinov, Viatcheslav A.Carrera Fernández, CeferinoMore, Swati S.Carresi, CristinaMoreno, J.J.Carrión, MarMoreno, Maria AngelesCarrizzo, AlbinoMoreno, Maria JoãoCarroll, MichaelMoreno, María U.Carrotta, RitaMoreno-Navarrete, José MaríaCarullo, GabrieleMori, KiyoshiCaruso, GianlucaMorillas Ruiz, Juana MariaCarvalho, Andreia N.Morimura, ShigeruCarvalho, CristinaMoriyama, YasukoCarvalho, DanielMorizane, RyujiCarvalho, PauloMorley, KristaCasanova, NancyMorris, KeithCasas, FrançoisMoruzzi, NoahCasas, Rosa M.Moschini, RobertaCascella, RaffaellaMoshage, HanCasili, GiovannaMoskovitz, JackobCasiraghi, AntonellaMot, AugustinCasola, AntonellaMotherway, Mary O’connellCassano, AlfredoMotohashi, KenCastagna, MichelaMotomura, NoboruCastellano, CarloMotoyama, KeiichiCastro-Caldas, MargaridaMottola, FilomenaCasuso, Rafael A.Mrakic-Sposta, SimonaCatalani, ElisabettaMuceniece, RutaCatani, Maria ValeriaMueller, JonathanCatar, RusanMulas, MaurizioCatarino, Marcelo D.Mulero, MiquelCaterina, ViglianisiMulet, Jose M.Catucci, GianlucaMüller-Deubert, SigridCauli, OmarMunakata, SatoruCaulin, CarlosMuntean, DaninaCavallaro, SebastianoMunteanu, Florenti-na-DanielaCavoski, IvanaMurakami, NaokaCazzola, RobertaMuraleva, Natalia A.Cebova, MartinaMurdaca, GiuseppeCecchi, LorenzoMure, KanaeCelano, RitaMurias, MarekCelec, PeterMurillo, Dra. María Luisa OjedaCenariu, MihaiMurugan, N. ArulCensi, SimonaMustonen, Anne-MariCerezo, MichaëlMuszyńska, EwaCerón, José JoaquínMuthukumaran, Jayara-manCerón-Carrasco, José P.Mutus, BulentCerrito, Maria GraziaMuzio, GiulianaCervellati, CarloMuzolf-Panek, MalgorzataCervellati, FrancoMuzolf-Panek, MałgorzataCervetto, ChiaraMyakala, KomuraiahCessna, StephenMylonas, AlessioChadjichristos, ChristosMytych, JenniferChadwick, Amy E.Nagata, EiichiroChae, HeeyoungNagata, NaotoChai, Tsun-ThaiNaiki-Ito, AyaChaki, MouniraNajlah, MohammadChalard, PierreNakamura, AkinobuChamcheu, Jean Christo-pherNakanishi, IkuoChan, CatherineNakazawa, MitsuruChan, Elsa C.Nalecz, HannaChan, JulieNalepa, IrenaChan, Shih-HungNalesso, FedericoChandrasekharan, DeepakNamgaladze, DmitryChang, Chia MingNanako, KawaguchiChang, Chia-cheNapoli, EleonoraChang, Chuang-RungNapolitano, AlessandraChang, Geng-RueiNapolitano, GaetanaChang, Hsueh-WeiNarayanan, S. PriyaChang, Jui-ChengNardi, MariateresaChang, Kee-lungNardi, SerenellaChang, Yuan-YenNaruishi, KojiChao, Louis KuopingNasim, Muhammad JawadChap, Hugues J.Nass, NorbertChapman, Katherine E.Nastasi, ClaudiaChapple, SarahNatalello, AntonioChartoumpekis, DionysiosNatarelli, LuciaChatre, LaurentNath, ArijitChatterjee, MadhumitaNathanailides, KosmasChauhan, BhareshNaujok, OrtwinChekanov, KonstantinNauser, ThomasChen, Bin-HueiNavarra, MicheleChen, Chien-LiangNavarro-Guillén, CarmenChen, Ching-YiNaviglio, DanieleChen, Chiung-MeiNaviglio, SilvioChen, Chun-JungNavrot, NicolasChen, JingNawirska-Olszańska, Ag-nieszkaChen, Liang-YuNawrot-Hadzik, IzabelaChen, MingshengNebija, DashnorChen, PingNedialkov, Paraskev TodorovChen, Po-HanNedyalkova, MiroslavaChen, Shih-HengNees, MatthiasChen, Shih-KuoNègre-Salvayre, AnneChen, Shuen-EiNeilson, AndrewChen, XiaoyanNeiva, Duarte M.Chen, XuqiaoNeunert, GrazynaChen, Yi-FanNeureiter, DanielChen, Yue-WenNeut, ChristelChénais, BenoitNevado, Paloma TejeraCheng, Hung-ChiNeves, DelmindaCheng, Juei-TangNgezahayo, AnacletCheng, Jya-WeiNguyen, Minh-ThuCheng, Pin-NanNicoletti, VincenzoCheng, RuiNicolini, GabriellaCheng, Yuan-BinNielsen, BrentCheong, Kit LeongNieva-Echevarría, BárbaraCherkaoui Malki, Mus-taphaNiimi, ManabuCherkaoui-Malki, Mus-taphaNikinmaa, MikkoChia, SeanNikolakaki, EleniChiavaroli, AnnalisaNikolova, GalinaChiba, HitoshiNiles, Richard MChimienti, Guglielmina AlessandraNinčević Grassino, An-tonelaChinnici, FabioNioi, ClaudiaChiodi, FrancescaNishi, KosukeChira, NicoletaNishijima, KazutoshiChirumbolo, SalvatoreNitulescu, MihaiChiummiento, LuciaNizza, AntoninoChizhov, Alexander O.Njus, DavidChizzola, RemigiusNobs, Samuel PhilipCho, JongkiNoce, AnnalisaCho, KyoungJooNocente, FrancescaChoi, Hyung-KyoonNociari, MarceloChoi, Jong-ilNogales, FátimaChoi, KihwanNoguchi, Constance TomChoi, Soo‐KyoungNogueira-Ferreira, RitaChou, Tzu-ChiehNogues, IsabelChristian Messerer, David AlexanderNolan, JohnChronopoulou, EvangeliaNoordergraaf, Inge-WillemChrysikou, LoukiaNorgren, LarsChu, Cheng-YingNota, AlessandroChuffa, Luiz GustavoNotas, GeorgeChung, SungjinNovikov, FedorChwil, MirosławaNowacka, MalgorzataChyau, Charng-CherngNowak, AnnaCianciolo, GiuseppeNowak, DariuszCiani, FrancescaNowak, DorotaCiarcia, RobertoNowak, RobertCiccarelli, MicheleNowicka, GrażynaCiccarese, FrancescoNowomiejska, KatarzynaCiccarone, FabioNozaki, MasamiCiccone, Marco MatteoNuc, KatarzynaCiccotosto, Giuseppe D.Nunes Da Silva, MartaÇiçek, Serhat SezaiNunes, AlexandraCicero, ArrigoNuñez, EstefaniaCichorek, MiroslawaNüssler, AndreasCichosz, MarcinNuţǎ, Diana CameliaCifelli, PierangeloNwaiwu, OgueriCioanca, OanaNycz, Jacek ECiobica, AlinNzwalo, HipolitoCione, ErikaOak, Min-HoCipak Gasparovic, AnaObana, AkiraČipak Gašparović, AnaObeng-Gyasi, EmmanuelCipak, LubosObri, ArnaudCires, EduardoOchi, ShinichiroCiriaco, FulvioOchmian, IreneuszCirillo, FedericaOczkowski, MichałCirmi, SantaOda, ShojiCisterna, BarbaraOdeh, MajedCiulla, MicheleOettmeier, ChristinaCiura, KrzesimirO'Flaherty, CristianCiz, MilanOh, JunseoCladis, DennisOhbayashi, NorihikoClanchy, FelixOhishi, TomokazuClaramunt, Rosa M.Ohnishi, MasatoshiClemens, MarkOhnishi, ShihoClement, AlbrechtOkumura, KoCobley, JamesOkumura, NobuakiCocco, RaffaellaOláh, AttilaCocco, StefaniaOláh, JuditCodella, RobertoOlah, Neli KingaCoelho, Sílvia CastroOlaru, Octavian TudorelCoïsson, Jean DanielOlaso, GloriaColantuoni, AntonioOlchawa, Magdalena M.Cole, MarshaOlchowik-Grabarek, EwaCollier, JasonOlczyk, KrystynaColucci, GiuseppeOlejnik, AnnaColumbano, AmedeoOliva, JoanColunga Biancatelli, Ruben Manuel LucianoOliveira, PedroCombaret, LydieOliver, EduardoConciatori, FabianaOliver, JordiConde, SilviaOliver, PeterConstantinescu-Aruxandei, DianaOliveri Conti, GeaContaldo, NicolettaOliviero, FrancescaContardi, MarcoOliviu, VostinaruConte, CarmelaOlsen, Lisbeth HøierConterato, Greicy M.Olson, KennethContreras, María Del MarOlszewska, Monika ACoombs, MelanieOmotehara, TakuyaCooper, KatrinaOndrias, KarolCopeland, PaulO'Neill, CatherineCopolovici, LucianOng, Eng ShiCoppock, Robert W.Onukwufor, John O.Coquerel, DavidOpazo, CarlosCör, DarijaOpiela, JolantaCordani, MarcoOprea, ElizaCordaro, MarikaOracz, JoannaCórdoba, ManuelOrian, LauraCorona, GiuliaOriente, FrancescoCorpe, ChristopherOristrell, JoaquimCorreia, MarceloOrlacchio, AntonioCorrell, NathanOrlandini, MaurizioCorso, GiovanniOroian, Mircea AdrianCosma, PinalysaOrso, EvelynCossignani, LinaOrtega, Angel L.Costa, Maria Do CéuOrtega, Joseph ThomasCosta, RobertoOrtega-Gutierrez, SilviaCostantini, LaraOrtolano, SaidaCostanzo, MicheleOrzolek, Michael D.Costanzo, PaolaOsmanović Barilar, JelenaCosta-Pinto, Ana RitaOsredkar, JoškoCoudert, JeromeOstrowski, RobertCoudray, CharlesOstróżka-Cieślik, AnetaCoué, MarineOta, ChiharuCoutinho, PaulaOthman, Eman M.Couturier, JeremyOtręba, MichałCravero, Maria CarlaOtsuka, YuzuruCrescente, GiuseppinaOvidi, ElisaCressatti, MarisaOwczarczyk-Saczonek, AgnieszkaCrestani, MaurizioOzaki, MichitakaCristina, Romeo T.Pace, PaulCroall, Iain DPacifico, SeverinaCroce, Anna CletaPaciolla, CostantinoCrosio, ClaudiaPaciotti, RobertoCross, NeilPage, MelissaCruz, Rui M.S.Pajares, MartaCruz-Chamorro, IvanPál, MagdaCséplő, ÁgnesPałasz, ArturCsiszár, JolánPalestini, PaolaCuadrado, MyriamPalikaras, KonstantinosCubero Pablo, María JoséPallàs Lliberia, MercèCullen, JosephPalleti Janardhan, HarishCulmsee, CarstenPalliyaguru, DushaniCulty, MartinePallotti, FrancescoCurreli, SabrinaPalmieri, ErikaCusanelli, EmilioPalmour, RobertaCwiklinski, KrystynaPalomba, LetiziaCzajkowski, RobertPalomo, Maria JesusCzerwinska, MonikaPan, Tai-longCzerwińska, MonikaPan, Yen-JuCzyż, JarosławPanaro, Maria AntoniettaCzyżowska, AgataPanasiewicz, GrzegorzD. Bortner, CarlPanayotova, VeselinaDa Silva, Anabela Ber-nardesPanfoli, IsabellaDaci, ArmondPang, Myung-GeolDaddam, Jayasimha Ray-aluPangrazzi, LucaDaferera, Dimitra J.Pansarasa, OriettaD'agostino, DonnaPantaleo, AntonellaDahl, Jan-UlrikPanzarella, VeraDailey, RobertPanzella, LuciaDalamaga, MariaPaolino, GiovanniDalefield, RosalindPaolucci, MarinaDalhaimer, PaulPapadopoulos, Elias G.Dallavalle, SabrinaPapadopoulos, GeorgiosDama, MuraliPapamatheakis, Joseph (Si-fis)Damiano, FabrizioPapież, MonikaD'Amico, RamonaPappa, AglaiaDamont, AnnelaureParađiković, NadaDamsteegt, Erin L.Paraskevaidis, IoannisDanac, RamonaParedes, FelipeDandlen, Susana AnahiPariente Llanos, Jose Anto-nioD’Angelo, StefaniaPark, Ah-MeeDarenskaya, Marina Ale-ksandrovnaPark, DaehoDario, BrunettiPark, Eun JeongDasiewicz, KrzysztofPark, Eun-YoungDavey, Pinakin GunvantPark, GunhyukDavid, Gabriela IuliaPark, Hang SooDavis, CatherinePark, Hyun JunDavis, IanPark, Jae-HyungDavison, GarethPark, Kyoung SikDaza-Navarro, PaulaPark, Kyoung-ChanD’Cunha, Nathan M.Park, Kyung-MokDe Bellis, LuigiPark, MinDe Benedetto, GiuseppePark, Sang WonDe Bruno, AlessandraParrella, EdoardoDe Deurwaerdere, PhilippeParsons, BarryDe Diego, Antonio M. G.Partyka, AgnieszkaDe Falco, BrunaPașca, ClaudiaDe Filippis, BarbaraPaśko, PawełDe Geest, BartPastore, AnalisaDe Guia, Roldan M.Pastorelli, GraziaDe Kock, JoeryPateiro, MirianDe La Torre, Maria ÁngelesPateras, Ioannis S.De Las Hazas, María Del Carmen LópezPatocs, AttilaDe Leonardis, AntonellaPatruno, AntoniaDe Luca, PaolaPattappa, GirishDe Luca, SimonePatti, AngelaDe Mario, AgnesePattillo, C.B.De Martin, SaraPattison, GrahamDe Rosa, Maria CristinaPaudel, RajDe Sousa-Coelho, Ana LuísaPaumann-Page, MartinaDe Stefani, DiegoPaventi, GianlucaDe Totero, DanielaPawlak, AleksandraDe Vita, DanielaPawlak, DariuszDe Vries, JanPawlas, NataliaDeb, SubrataPawlik, AndrzejDe-Bandt, Jean-PascalPazdro, RobertDecker, PatricePeana, Massimiliano F.Dedoni, SimonaPearce, MargaretDegl'innocenti, DonatellaPec, MartinDel Bo', CristianPech-Cervantes, Andres AlfredoDel Casale, AntonioPedraza Chaverri, JoséDel Vecchio, AlessandroPeer, CodyDeli, CharikliaPeh, Hong YongDeliyanti, DevyPehar, MarianaDella Morte, DavidPeinado, María ÁngelesDemiot, ClairePeixoto, FranciscoDemonacos, ConstantinosPeleli, MariaDemopoulos, Constantinos AlexandrosPelidou, Sygkliti-HenriettaDémuth, BalázsPellegrini, MarikaDeochand, DineshPellegrini-Giampietro, Domenico EdoardoDepeint, FlorePellitteri, Rosalia Maria CristinaDesfontis, Jean ClaudePeluso, IlariaDeslandes, EricPeluso, Marco E. M.Desmarchais, AlicePenke, LokaDesogus, FrancescoPenna, ClaudiaDetsika, Maria G.Pennarun, GaëlleDev, SomPenning, LouisDevkota, Hari PrasadPepe, SalvatoreDevlin, Angela M.Peragón, JuanDey, PriyankarPercival-Smith, AnthonyDi Conza, GiusyPerdomo, GermanDi Franco, SimonePereira, Antía GonzálezDi Giacomo, ClaudiaPereira, Carlos DiasDi Giulio, CamilloPereira, DavidDi Lorenzo, FrancescoPereira, ElianaDi Marco, GabrielePereira, LeonelDi Maro, AntimoPereira, Maria De LourdesDi Martino, CatelloPereira, Patrícia AlexandraDi Nunzio, MattiaPereira, PaulaDi Pietro, Maria EnricaPereira-da-Silva, LuisDi Pietro, NataliaPerestrelo, RosaDi Salle, AnnaPérez, SalvadorDi Sanzo, RosaPérez-Gálvez, AntonioDi Simone, NicolettaPerez-Pe, RosauraDi Sotto, AntonellaPeris, José-EstebanDi Stefano, AntonioPerkins, AndrewDiamond, AlanPerła-Kaján, JoannaDiamond, GillPeron, GregorioDickinson, Jared MPerrelli, AndreaDidangelos, TriantafyllosPerrino, Enrico VitoDikalova, Anna EPerrone, SerafinaDimarogona, MariaPerrotta, Ida DanielaDimauro, IvanPers-Kamczyc, EmiliaDimienescu, Oana GabrielaPerut, FrancescaDimitrov, IvanPes, GianniDini, PouyaPeskin, AlexanderDinică, RodicaPessina, FedericaDinica, Rodica-MihaelaPessler, FrankDinis, Lia Tânia JPeters, MarcusDinischiotu, AncaPetersen, ElijahDipnall, JoannaPetit, Patrice X.Dludla, Phiwayinkosi VusiPetrie-Hanson, LoraDo, BernardPetrivalsky, MarekDobrikova, AneliaPetrotos, KonstantinosDobrowolski, PiotrPetrova, GuenkaDolce, DanielaPetruczynik, AnnaDolivo, DavidPetry, AndreasDołowy, MałgorzataPeulen, OlivierDomin, HelenaPey, Angel L.Domoki, FerencPhan, Thi Tuong VyDonakonda, SainitinPhelix, Clyde F.Donato, PaolaPiacente, SoniaDonato, RosarioPiasecka, MałgorzataDonato, Rosario FrancescoPiątczak, EwelinaDong, BoPiątkowska, EwaDonnini, SandraPiccinin, ElenaDordevic, DaniPiccionello, Antonio Pa-lumboDoria, EnricoPiccoli, MarcoDorszewska, JolantaPick, EdgarDos Santos, Regiane Ro-driguesPicone, PasqualeDosche, CarstenPiechota-Polanczyk, Ale-ksandraDosoky, NouraPiedade, Ana PaulaDossena, SilviaPiemonte, FiorellaDou, JinzePieniazek, AnnaDowning, JeffreyPieri, MassimoDrabińska, NataliaPierzchala, KatarzynaDraganidis, DimitriosPietrangelo, LauraDragon-Durey, Ma-rie-agnesPietrowska-Borek, Małgor-zataDragonieri, SilvanoPignataro, GiuliaDražić, MajaPignitter, MarcDrenjančević, InesPiiper, AlbrechtDrevet, Joel R.Piknova, BarboraDrillich, MarcPiłsyk, SebastianDrozd, RadoslawPin, FabrizioDruzynska, BeataPinart, ElisabethDrużyńska, BeataPincemail, JoëlDuarte, DelfimPinchuk, IlyaDuarte, Filipe V.Piñeiro, AngelDubos, ChristianPinela, JoséDucatelle, RichardPiñero, José EDucreux, LaurencePinheiro, JoaquinaDudas, JozsefPini, Luigi AlbertoDudová, IvaPinotti, LucianoDuennwald, Martin LPinto, LuísDuguez, StephaniePintus, GianfrancoDulf, FranciscPiomboni, PaolaDumancas, GerardPirina, PietroDumnicka, PaulinaPirkmajer, SergejDunkić, ValerijaPirola, LucianoDunlop, RachaelPiscopo, MarinaDuraccio, DonatellaPizent, AlicaDurán-Prado, MarioPlant, LeighDurante, MirianaPlastina, PierluigiDuranti, GuglielmoPlatania, ChiaraD'Uscio, Livius VPlatta, HaraldDutartre, PatrickPlatta, Harald W.Dutta, RajeshPlaza-Sirvent, CarlosDutu, LigiaPŁażek, AgnieszkaDuvigneau, J. CatharinaPłotka-Wasylka, JustynaDuvigneau, Johanna Cath-arinaPlotnikov, EgorDvorakova, Magdalena ChottovaPobiega, KatarzynaDymkowska, DorotaPogacnik, LeaDyshlovoy, Sergey A.Poiana, Mariana-AtenaDzidic, AlenPokora, IlonaDziekońska, AnnaPokrywka, AndrzejDzika, EwaPolčic, PeterDziki, DariuszPolge, CécileDżugan, MałgorzataPolicar, ClotildeE. Golub, IgorPöling, JochenEamens, Andrew L.Polissidis, AlexiaEbara, MitsuhiroPoljšak, BorutEccher, AlbinoPolz-Dacewicz, MałgorzataEdge, RuthPomastowski, Paweł P.Edmunds, Lia R.Pomierny, BartoszEduardo-Figueira, MariaPon Velayutham, Anandh BabuEfthimia, CotouPond, Amber L.Egawa, TatsuroPonsonby, Anne-LouiseEggler, AimeePoole, Leslie B.Eisenreich, AndreasPopescu, Mihaela R.El Bounkari, OmarPopescu, SilvanaElchaninov, AndreyPopova, TatyanaElena, EnachiPorro, BenedettaElida Nora, FerriPósa, AnikóElKelish, AmrPospisil, JiriElsner, MatthiasPotaczek, Daniel P.El-Sohaimy, SobhyPoteser, MichaelEndo, MakotoPotočnjak, IvaEndre Károly, KristófPotrykus, KatarzynaEngevik, KristenPouget, ChristelleEnoki, YukiPoulsen, KyleEnosawa, ShinPoupot, RemyEom, Gwang HyeonPourié, GrégoryEpping, Eric A.Pourzand, ChararehErjavec, Gordana NedicPowell, Folami LamokeErland, LaurenPrasad, Kedar N.Ermolenko, Ermolenko E.I.Pratesi, AlessandroErrigo, AlessandraPrats, María SoledadEsatbeyolgu, TubaPrieto, IsabelEscames, GermainePrieto, MartaEscolà-Gil, Joan CarlesPrieto, Miguel A.Escudero, OlgaPrieto, Miguel ÁngelEskin, MichaelPrieto, Rafael MariaEskiw, Christopher H.Prigge, Justin R.Esnault, Vincent L.M.Prignano, FrancescaEspinosa, FranciscoPrimavera, RositaEsposito, PasqualePrimiano, GuidoEsteban, MaríaPripp, Are HugoEzhov, MaratPrkić, AnteFabiani, RobertoPrlić Kardum, JasnaFabrizi, CinziaProestos, CharalamposFabrizio, MontecuccoPronin, Alexander V.Fadda, AngelaProsperi, EnnioFagerstedt, KurtProsperi, JeniferFagone, PaoloProtchenko, OlgaFalcão, Soraia I.Puigdomènech, PereFalcone, ItaliaPukalskas, AudriusFang, Evandro FeiPula, GiordanoFarcasanu, Ileana C.Py, BeatriceFarkas, ÁgnesQiu, BinFarooq, ShahidQuaglino, DanielaFasler-Kan, ElizavetaQue, LongFasolato, LucaQuigley, John GFasolino, InesQuindry, JohnFassl, AnneRab, AndrásFauconnier, Marie LaureRabanel, Jean MichelFavier, JudithRabe, EberhardFedoreyev, SergeyRaboni, SamantaFeito, JorgeRacay, PeterFeldo, MarcinRachek, LyudmilaFelice, RobertoRacz, AttilaFelix, KlausRaczyńska, EwaFeng, Chia-HsienRadak, ZsoltFerenc, PajorRadonić, AniFeresin, Rafaela G.Radu, NicoletaFernadez-Bolanos, José G.Ragazzi, EugenioFernandes, ElisabeteRaggi, CarlaFernandez De Simon, BrigidaRagni, MaurizioFernandez, AdelaideRahimi, FaridFernandez, CesarRaji, IdrisFernandez, MercedesRajoka, Muhammad Sha-hid RiazFerramosca, AlessandraRamalho-Santos, JoãoFerrarese, CarloRamanauskienė, KristinaFerreira, NelsonRamírez, JuliaFerreira, Nuno R.Ramljak, JelenaFerreira, Paula M.Rammes, GerhardFerreira-Santos, PedroRamos, EvaFerreiro-González, MartaRamos, Marco A.Ferrero, Maria ElenaRamos, Patricia A.B.Ferri, NicolaRamos, PawełFerro, MatteoRankin, GaryFielitz, JensRanstam, JonasFierascu, IrinaRanzato, EliaFigueiredo, Ana CristinaRao, GovindFiligheddu, NicolettaRapacz, MarcinFilip, RafałRapala-Kozik, MariaFilipek, AgnieszkaRapeanu, GabrielaFilipek, AnnaRaptis, Athanasios E.Filipovic, Milos RRasola, AndreaFin, AndreaRastogi, AnshuFini, Mehdi ARaudonė, LinaFinicelli, MauroRebollo-Hernanz, MiguelFiorito, FilomenaRegensburger, MartinFirczuk, MalgorzataRegiec, AndrzejFischer-Fodor, EvaRengo, SandroFisher, Aron B.Requejo-Aguilar, RaquelFitzGerald, RexRescigno, AntonioFlora, GaganReshkin, StephanFlorek, MariuszRetta, Saverio FrancescoFogacci, FedericaReuter, KlausFolco, Hernan DiegoRey, PascalFoligni, RobertaReybier, KarineFonseca, Bruno Miguel Reis DaReynaert, Niki LFontenla, Jose AngelRha, Chan-SuFontés, MichelRibeiro, ArturForesti, RobertaRicci, AriannaForini, Francesca S.Ricci, GiorgioFornai, FrancescoRicci, GiuseppeForte, ElenaRiccio, GennaroForte, MaurizioRiccò, MatteoForti, LucaRichaud, EmmanuelForzato, CristinaRiessland, MarkusFrancés, RubénRigano, DanielaFrancesco, AnielloRijo, PatríciaFranchin, CinziaRinnerthaler, MarkFranco, AntoniettaRipoli, CristianFrançois, CasasRishi, ArunFranzoni, FerdinandoRius-Pérez, SergioFreire, M. SoniaRiuzzi, FrancescaFreitas De Freitas, LucasRivera Del Álamo, Maria MontserratFrench, CurtisRives, NathalieFreschi, PierangeloRizzarelli, EnricoFriedman, HayaRizzi, LauraFrolíková, MichaelaRizzo, AngelaFrolov, AndrejRizzo, GianlucaFrontiñán-Rubio, JavierRizzolio, FlavioFrosini, MariaRoca, JordiFu, JieRocca, CarmineFu, QinRocca, RobertaFuentes, Jose ManuelRocchiccioli, SilviaFujimori, KoRocco, LuciaFujiwara, KeigiRoche, EnriqueFukuda, MitsuruRochfort, KeithFukumoto, YoshihiroRockenfeller, PatrickFukushima, AtsushiRodrigues, Andreia C. M.Fulignati, SaraRodrigues, FranciscaFumiaki, ItoRodrigues, MárcioFunaki, MakotoRodrigues, Márcio José De AbreuFunk, RichardRodrígues-Díez, RaquelFurdui, BiancaRodríguez Arcos, María RocíoFuruichi, KengoRodriguez, GuillermoGabbia, DanielaRodriguez, JoseGabbianelli, RositaRodriguez-Vargas, Jose ManuelGabriele, MorenaRodríguez-Yoldi, María JesúsGabrielli, Maria GabriellaRodriquez, AnaGaburjáková, MartaRoduit, RaphaelGad, AhmedRogobete, Alexandru FlorinGafencu, AncaRoh, SanghoGaffke, LidiaRohde, DavidGagaoua, MohammedRoj, EdwardGagat, PrzemyslawRój, EdwardGai, FrancescoRojas, ArmandoGajęcki, MaciejRojo, Jose M.Gajek, ArkadiuszRok, JakubGajewska, MalgorzataRolla, SimonaGajos-Michniewicz, AnnaRoma, JoanaGal, Adrian FlorinRomani, AndreaGalán, Miguel LaderoRomano, Giovanni LucaGalanty, AgnieszkaRomero, Francisco JavierGalardon, ErwanRonavari, AndreaGalaris, DimitriosRondanino, ChristineGalatou, EleftheriaRong, ShisongGalbiati, MariaritaRoot, MartinGalindo, Máximo IboRos Berruezo, GasparGaliniak, SabinaRosa, AntonellaGallar, JuanaRosado, CarlosGallart Ayala, HectorRosado, Iván ValleGallazzini, MorganRoșca, Mariana G.Galleri, GraziaRosenberg, MosheGalli, FrancescoRosenthal, KatrinGallo, Maria PiaRossi, RaffaellaGallorini, MarialuciaRossin, DanielaGallyas, FerencRosso, ChiaraGalter, DagmarRostoker, GuyGalvan, Daniel LRoszak, JoannaGalvano, FabioRoth, MichaelGamberucci, AlessandraRotllan Vila, NoemiGamon, Luke FrancisRotti, PavanaGan, RenyouRoubelakis-Angelakis, Kal-liopi A.Gancarz, RomanRouhier, Matthew F.Ganesan, A.Rouleau, MatthieuGantner, MagdalenaRoumeliotis, StefanosGao, ChunqiRoumenina, LubkaGarbayo, InésRoumes, HélèneGarcía Gonzalo, FrancescRoverso, MarcoGarcía, José JoaquínRovito, DanielaGarcia, LorenzoRóżycki, MirosławGarcia, MagdalenaRuaro, BarbaraGarcía, MontserratRubilotta, EmanueleGarcia-Alonso, AlejandraRubino, Federico MariaGarcía-Corzo, LauraRubinowska, KatarzynaGarcía-Estañ, JoaquínRucińska-Sobkowiak, Re-nataGarcia-Gil, MercedesRucker, Robert B.Garcia-Granda, SantiagoRudzińska, MagdalenaGarcía-Herrera, PatriciaRufino, AnaGarcía-Pastor, María E.Ruiu, RobertoGarcía-Rodríguez, AserRuiz, AinhoaGardner, DavidRuiz-Gómez, Miguel J.Garella, RacheleRuiz-López, Mario AlbertGariboldi, ManuelaRussell, FraserGarozzo, DomenicoRussell, MarkGáspár, RóbertRusso, IsabellaGasperi, ValeriaRusso, Mario VincenzoGates, ColinRuvio, RaquelGavaldà-Navarro, AleixRybak, KatarzynaGavriilaki, EleniRybczyńska-Tkaczyk, Ka-milaGavrila, Adina IonutaRyffel, BernhardGaweł-Bęben, KatarzynaRyu, ChangseonGazzin, SilviaRzemieniec, JoannaGeen-Dong, ChangRzepecka-Stojko, AnnaGęgotek, AgnieszkaSabater, BartolomeGekle, MichaelSachivkina, NadezhdaGeloso, Maria ConcettaSachmechi, IssacGelzo, MonicaSack, UlrichGenders, AmandaSaeed, AliGennaro, RenatoSáenz-Medina, JavierGenova, Maria LuisaSaéz, Juan CarlosGenovese, IlariaSahoo, Bikash R.Gentile, FabrizioSahrawy, MariamGeorgescu (Serb), AlinaSaini, Ramesh KumarGere, AttilaSajeva, MaurizioGerić, MarkoSak, Jarosław J.Gericke, AdrianSakakibara, HiroyukiGermana, AntoninoSakamoto, KojiGerothanassis, Ioannis P.Sakamoto, TakuyaGesek, MichałSakasai-Sakai, AkikoGhassemi Nejad, JalilSakayori, NobuyukiGhelardoni, SandraSako, KaoriGhezzo, AlessandroSakuma, KunihiroGhiasi, Seyed MojtabaSakurada, KazuhiroGhilardi Lago, João Hen-riqueSala, GessicaGhobadloo, ShahrokhSalafranca Lázaro, Fran-cisco J.Ghosh, SubhajitSalaroli, RobertaGi, SangBaeSalat, KingaGiacomazza, DanielaSalcedo, InmaculadaGiakoustidis, Dimitris E.Saleh-E-In, Md. MoshfekusGiampieri, FrancescaSalifoglou, AthanasiosGiampietro, LetiziaSalman, MootazGiebultowicz, JoannaSalopek-Sondi, BrankaGieczewska, KatarzynaSalucci, SaraGierszewska, MagdalenaSalvador, ArmindoGigliotti, Casimiro LucaSalvo, AndreaGil-Solsona, RubénSambri, AndreaGiménez, MaríaŠamec, DunjaGiménez-Bastida, Juan AntonioSamhan-Arias, AlejandroGiner-Tarrida, LuísSamiec, MarcinGinsberg, Stephen D.Sammarco, GiuseppeGiordano, PaolaSampaio-Marques, BelémGiovannelli, LisaSampedro, DiegoGiovannelli, PiaSánchez López, ElenaGirard, Pierre-MarieSánchez-Escalante, ArmidaGismondi, AngeloSancho, PatriciaGiudice, Giuseppe LoSandbichler, AdolfGiuffrè, Angelo MariaSangenito, Leandro StefanoGiuffrida, DanieleSanmartín Grijalba, Car-menGiuliano, Michela M.San-Miguel, BeatrizGiuseppina, BasiniSanna, GavinoGiustarini, DanielaSansone, AnnaGiusti, LauraSantacroce, LuigiGlantzounis, Georgios K.Santanam, NaliniGlickman, MichaelSantangelo, CarmelaGlogowska, AleksandraSantarpino, GiuseppeGluhovschi, CristinaSantilio, AngelaGniazdowska-Piekarska, AgnieszkaSantoro, MassimoGo, Gwang-WoongSantos López, Jorge ArturoGodlewska, MarlenaSantos Nascimento, Luana Beatriz DosGodos, JustynaSantos, Carla S.Goetten De Lima, GabrielSantos, RenatoGokulan, Ravindran CaspaSantos-Ocaña, CarlosGołąbek-Sulejczak, DorotaSantulli, GaetanoGold, MatthewSaponaro, ConcettaGoldoni, MatteoSaratale, Ganesh Datta-trayaGoldstein, SaraSardo, AngelaGołembiowska, KrystynaSarecka-Hujar, BeataGolinelli, Marie-PierreSaretzki, GabrieleGómez Gómez, FelipeSarsour, Ehab H.Gomez, JesusSassoli, ChiaraGómez-Torres, Maria JoséSastre-Serra, JorgeGoni, FelixSato, KazuomiGonon, GeraldineSato, ReiichiroGonthier, Marie-PauleSaul, NadineGonzález Laredo, Rubén F.Savic, Ivan M.Gonzalez Noya, AnaSavion, NaphtaliGonzalez-Bulnes, AntonioSavu, AnamariaGonzález-De-Peredo, Ana V.Sawicka, BarbaraGonzalez-Garcia, Maria J.Sayago, AnaGonzalez-Menendez, PedroSazonova, Margarita A.González-Mesa, ErnestoScampicchio, MatteoGonzález-Minero, Francis-co JoséScarafoni, AlessioGonzalez-Navarro, Her-miniaScatena, RobertoGonzález-Nieto, DanielScavuzzi, Bruna MiglioranzaGonzález-Rodríguez, Ma-ría LScharf, MichaelGopal, KeshavScharnagl, HubertGordijn, Sanne J.Scheau, CristianGordon, Bradley S.Schechter, Alan NeilGoričar, KatjaScheinman, RobertGorniak, PatrykSchilling, JoelGórska, AgataSchipke, Jochen DGorynski, KrzysztofSchirmer, BastianGoto, KatsumasaSchlicker, EberhardGoto, KeiSchling, PetraGotoh, HiroakiSchlüter, Klaus-DieterGougoulis, Dimitris A.Schmalhausen, E.V.Grabarczyk, MałgorzataSchmitt, FelixGradinaru, RobertSchneider, JochenGramsbergen, JanSchönbeck, Jens Christian SidneyGramza – Michałowska, AnnaSchorr, Mark S.Granica, SebastianSchröder, KatrinGranquist, Erik GeorgSchupp, NicoleGrasa, LauraSchwans, Jason P.Graulet, BenoîtSchwarzinger, BettinaGravato, CarlosSciandra, FrancescaGray, Nora EScibior, AgnieszkaGreco, PantaleoŚcibisz, IwonaGreenlief, C. MichaelScicchitano, Bianca MariaGreinert, RüdigerŚciskalska, MilenaGrela, Eugeniusz RyszardSciubba, FabioGrembecka, MałgorzataScoditti, EgeriaGriesbeck, Axel G.Sęczyk, ŁukaszGrigoriev, I. P.See Hoe, Louise E.Grilli, AlfredoSegatto, MarcoGrillo, GiorgioSeiquer, IsabelGrimm, Marcus OWSellars, JonathanGrocholewicz, KatarzynaSelvaraj, GurudeebanGromadzka-Ostrowska, JoannaSenbonmatsu, TakaakiGronewold, JanineSenin, IvanGrossini, ElenaSeo, Young-KwonGrosso, ClaraSeoane-Collazo, PatriciaGrosso, GiuseppeSeong, Ki MoonGruhlke, Martin C. H.Serefko, AnnaGrumati, PaoloSerefko, Anna D.Guarino, CarmineSerezhenkov, VladimirGubal, AnnaSergeeva, ElenaGubitosa, JenniferSerpico, RosarioGude, Natalie A.Serratosa, Maria P.Guerra, FloraSertic, SarahGuerrieri, NicolettaSessa, FrancescoGuevara-Lora, IbethSethi, GautamGugliuzza, GiovanniSetzer, William N.Guibert, ChristelleSevastre, BogdanGuidi, PatriziaSeverino, PaoloGuille, MattSevilla-Morán, BeatrizGuillén Viejo, CarlosSgarbossa, AntonellaGuillén-Bejarano, RafaelSgherri, CristinaGuillén-Sánchez, Dominico AShabtay, AyeletGuix, Francesc XavierShadfar, SinaGulya, K.Shankar, KripaGuo, Chih-HungSharapov, Mars GGuo, JinhuSharma, ArpeetaGupta, RajeshSharman, Edward H.Gupta, RaviSherwani, Mohammad As-ifGuriec, NathalieShieh, Jeng-jerGutierrez-Mariscal, Fran-cisco M.Shikama, YosukeGvozdjaková, AnnaShimasaki, YouheiHa, EunyoungShimizu, KaorukoHa, Jung-HeunShin, Chan YoungHadjikakou, SotirisShirato, KenHafstad, Anne DragøyShoda, MakotoHägglund, Per MårtenShukla, KirtikarHaidl, GerhardShults, Elvira E.Haigh, AnthonyShults, Nataliia V.Haines, Julie ReiszShurubor, Yevgeniya I.Hajdari, AvniSi, HongweiHajduch, EricSicińska, PaulinaHajieva, ParvanaSidoti, AntoninaHallmann, EwelinaSienkiewicz, MonikaHamano, TadanoriSignorile, AnnaHamczyk, Magda R.Sikazwe, DonaldHammer, AstridSilaghi-Dumitrescu, RaduHammill, JaredSilva Moura, Elisabete Fer-reiraHammond, Billy R.Silva, Célia C.G.Hampton, Mark B.Silva, Marcelo SousaHan, Yeon SooSilva, PaulaHanba, Yuko T.Silva, RuiHancu, GabrielSilvagno, FrancescaHannani, DalilSilvestre, Miguel AngelHannibal, LucianaSilvestri, CristianHansíková, HanaSilvestrini, AndreaHantusch, BrigitteSimão, André L.Hao, FuhuaSimitzis, PanagiotisHaque, Md. MamunulSimm, StefanHara, HiroshiSimonini, SaraHara, ShuntaroSimpson, AlecHarashima, NanaeSinger, GeorgHardacre, AllanSingh, Anand PrakashHargreaves, IainSingh, HarpalHargreaves, Iain ParrySingh, KarmveerHarman, DavidSiniscalco, DarioHarvey, AlexandraSipos, KatalinHasegawa, MorifumiSips, PatrickHashimoto, MakotoSirangelo, IvanaHashimoto, YosukeSiristatidis, CharalamposHassan, Sherif T.S.Sisalli, Maria JosèHässig, MichaelSisti, DavideHatano, TsutomuSjoholm, AkeHatori, YutaSkała, EwaHawkes, DavidSkånland, Sigrid S.Haybaeck, JohannesSkarżewski, JacekHaynes, JenniferSkenderidis, ProdromosHazai, LászlóSkoczylas, ŁukaszHedrich, SabrinaSkorupa, MonikaHegazy, Mohamed-Elamir F.Skórzyńska-Dziduszko, KatarzynaHeim, BeatriceSkouta, RachidHeleno, SandrinaSkroza, DanijelaHenkel, JaninSkrypnik, LiubovHenríquez-Olguín, CarlosSkrypnik, Liubov N.Heras Benito, ManuelSkrzep-Poloczek, BronisławaHermesz, EditSlape, Christopher I.Hernansanz-Agustín, PabloSlavik, LudekHerrera, Antonio J.Sliwinska, AgnieszkaHerrera, FedericoSłomińska-Wojewódzka, MonikaHerrera-Molina, RodrigoSlováková, ĽudmilaHerrero, EnriqueSmeriglio, AntonellaHerrero, María JesúsSmirnoff, NicholasHershfinkel, MichalSmolarczyk, RyszardHervé, TécherSmykal, PetrHeusch, GerdSneddon, JenniferHickey, MiriamSnider, Ashley JHidalgo, ElenaSoares, CristinaHier, DanielSoares, PedroHiguchi, KeiichiSobierajska, KatarzynaHildenbrand, GeorgSocea, BogdanHilhorst, MarcSocha, RobertHirano, KatsuyaSokovic, MarinaHirasawa, NoriyasuSolano, FranciscoHirotaka, KanedaSoleti, RaffaellaHo, Jar-YiSolimando, Antonio G.Hodges, MichaelSomlyai, GáborHoffmann, Klaus H.Sommaggio, DanieleHögman, MarieannSommer, SylwesterHolatko, JiriSon, Chang GueHolmes, Andrew PSong, JiajiaHolmes-Hampton, GregorySonntag, KaiHomma, KoheiSonowal, HimangshuHomman-Ludiye, JihaneSorg, OlivierHong, Jiann-RueySortino, Maria AngelaHonsho, MasanoriSosic, IzidorHopfer, HeleneSotomayor, Camilo G.Horhat, DeliaSottero, BarbaraHornick, Jean-LucSousa, Maria JoãoHorowitz, John D.Sovadinová, IvaHorváth, Eszter MáriaSoveral, GraçaHosseinkhani, HosseinSowa, IreneuszHou, Chih YaoȘpac, AdrianHouhoula, DimitraSpadaro, AngeloHrabia, AnnaSpadaro, SavinoHsia, Shih-MinSpagnuolo, CarmelaHsieh, Lu-ShengSpann, KirstenHsieh, Ming-JuSpasov, Alexander A.Hsieh, Tusty-JuanSpizzirri, U GianfrancoHsu, Chin-LinSprícigo, José Felipe Warm-lingHsu, Chung Y.Spyroglou, AriadniHsu, Kai-WenStacchiotti, AlessandraHsu, Tsai-ChingStadnyk, AndrewHsu, Yu-ChiaStahl, Chad H.Hu, BinStan, LauraHuang, ChunlingStančiaková, LuciaHuang, Chun-YungStanek, AgataHuang, Guan-JhongStarobova, HanaHuang, Huey-ChunStarowicz, MałgorzataHuang, Hui-ChunStarzyński, RafałHuang, Shih-YiStaszczak, MagdalenaHuang, Wei-LunStătescu, CristianHuang, XudongStateva, RoumianaHudemann, ChristophStathopulos, PeterHuertas, Jesús R.Stec, David E.Huh, Joo YoungStefanucci, AzzurraHulina-Tomašković, An-dreaStehle, PeterHume, Michael E.Steinbrenner, HolgerHung, Shih-YaStemmler, MarkHuppi, KonradStępień, Agnieszka EwaHurtado-Barroso, SaraStepien, Karolina M.Hussain, HidayatStępniowska, AnnaHussain, MulazimStoian, Anca Mihaela PanteaHwang, Dae YounŠtolfa, IvnaHwang, HeeyounStorsberg, JoachimHybertson, Brooks M.Stoyanova, AlbenaHytti, MariaStraface, ElisabettaHyun, Sang-HwanStraub, KarlIampietro, MathieuStrelec, IvicaIanni, FedericaStrzemski, MaciejIciek, MałgorzataStudzinska-Sroka, ElzbietaIdoate, MiguelStuper-Szablewska, KingaIentile, RiccardoStushnoff, CecilIevinsh, GedertsStutz, BernardoIkezumi, YoheiSubramani Paranthaman, BalasubramaniIlia, GheorgheSuddala, Krishna ChaitanyaIlic, NebojsaSugimoto, MasayukiImamura, MasahiroȘuică-Bunghez, RalucaImperatore, RobertaSulej, JustynaImran, SaimaSuliman, Hagir BInayathullah, MohammedSullivan, RyanIndra, RadekSun, CongshanInoue, HirofumiSun, RamonIonescu, Elena RodicaSurai, Peter F.Ionita, PetreSuravajhala, PrashanthIovieno, PaoloSutton, Adam T.Irakli, MariaSuzuki, NaoIrisawa, AtsushiSvoronos, Paris D. N.Ishihara, KatsuhikoSykiotis, Gerasimos P.Ishikawa, KiyotakeSykłowska-Baranek, KatarzynaIshino, YoshizumiSynytsya, AndriyIskra, MariaSytar, OksanaIslam, Md SorifulSzabo, IldikoIsola, GaetanoSzabo, KatalinIssagouliantis, MariaSzaleniec, MaciejIto, JunyaSzanto, IldikoIto, TetsufumiSzczepaniak, OskarItoh, KenSzczepek, Agnieszka J.Iuliano, RodolfoSzeiffova Bacova, BarbaraIvic, IvanaSzewczyk, KatarzynaIwai, AtsushiSzewczyk-Golec, KarolinaIwaoka, MichioSzopa, AgnieszkaIwaszkiewicz-Grzes, DorotaSztanke, KrzysztofIzawa, Kazuhiro P.Sztanke, MałgorzataIzdebska, MagdalenaSzulc-Dąbrowska, LidiaIzquierdo-Rico, Maria JoséSzumiło, JustynaJaburek, MartinSzutowicz, AndrzejJacob, Maik H.Szwajca, AnnaJacobo-Velazquez, DanielSzweda, PiotrJacomin, Anne-ClaireSzwengiel, ArturJafri, MohsinSzychowski, Konrad A.Jaganjac, MoranaSzymandera-Buszka, KrystynaJaggers, JasonSzymańska, RenataJais, AlexanderTaddei, Maria LetiziaJakobek, LidijaTakemori, HiroshiJakobusic Brala, CvijetaTakeuchi, HideyukiJakovac, HrvojeTalora, ClaudioJakovljevic, MartinaTamasi, GabriellaJakubczyk, AnnaTamba, Bogdan IonelJakubczyk, EwaTambuwala, MurtazaJakubczyk, KarolinaTampa, MirceaJaleel, AbdulTan, Bee KJames, JoelTanaka, HajimeJames, Nicholas G.Tanaka, JunichiJańczak-Pieniążek, MartaTanaka, NaoroJanda, ElzbietaTanase, CorneliuJanda, KatarzynaTao-Cheng, Jung-HwaJandeleit-Dahm, KarinTarantilis, PetrosJaniszewska-Turak, EmiliaTarasco, EustachioJanja, KristlTarcea, MonicaJanssen, BienekeTarrago, LionelJantas, DanutaTattini, MassimilianoJara Palacios, María JoséTatullo, MarcoJarcuska, PeterTavazzi, BarbaraJarzebski, MaciejTayebati, Seyed KhosrowJasińska-Stroschein, Mag-dalenaTchetina, Elena V.Jastrzebska, BeataTedeschi, PaolaJavadov, SabzaliTedesco, IdoloJaworek, Andrzej Kazimi-erzTeixeira, NatérciaJazvinšćak Jembrek, MajaTejero, JesúsJe, Jae-YoungTen Have, GabriellaJean, ChristineTenenbaum, LilianeJeandet, PhilippeTenopoulou, MargaritaJedrejek, DariuszTervaert, Jan Willem CohenJelcic, IlijasTeschke, RolfJeleń, MałgorzataTeter, KenJembrek, Maja JazvinšćakThackray, AlanaJena, Prasant KumarTheilmann, LorenzJeong, Seon-YongTheiss, CarstenJeongjin, ParkThérèse Charles, MarieJerković, IgorThiruvengadam, MuthuJeromel, AnaThomas, John C.Jesudhasan, PalmyThomas, Roger E.Jeszka-Skowron, Magdale-naThorenoor, NithyanandaJethava, Krupal P.Thuillier, RaphaelJiang, Shu-YeTian, QiyuJiménez, AnaTiano, LucaJiménez-Araujo, AnaTiberio, LauraJiménez-Monreal, Antonia M.Tibullo, DanieleJin, Byung-KwanTiidus, PeterJin-chul, KimTiirikainen, MaaritJo, Min GiTijssen, PeterJobling, MalcolmTimoshenko, Alexander V.Jodynis-Liebert, JadwigaTimpani, CaraJohn Matthew, MahoneyTirillini, BrunoJoles, Jaap A.Tisi, RenataJones, Meriel G.Tlak Gajger, IvanaJong, Chian JuTobiszewski, MarekJong, ChianjuTodaka, DaisukeJonscher, Karen R.Todisco, SimonaJoo, Jae-YeolTojo, ConchaJordao, LuisaTokarz, PaulinaJörns, AnneTokuda, MasaharuJosé Tauler Riera, PedroToledano, Michel B.Josef, KapfhammerToma, LauraJoseph, Leroy C.Tomasello, Marianna FloraJoshi, SuchitraTomaszewska, EwaJozkowicz, AlicjaTomaszewska-Gras, Jolan-taJug, BorutTong, JunchaoJukić Špika, MajaTony, HaighJullian, NathalieTooyama, IkuoJumbo-Lucioni, PatriciaTopunov, Alexey F.Jung, HeewonTorras-Ambròs, JoanJung, JunyangTorre, Maria LuisaJung, Sung KeunTorres Fuentes, CristinaJung, Young SungTorres-Sánchez, IreneJungnickel, ChristianTósaki, ÁrpádJurášek, MichalTošović, JelenaJurić, SlavenTotan, Alexandra RipszkyJurisicova, AndreaToti, PaoloJuśkiewicz, JerzyTotta, PierangelaJuszczak, LesławTouraud, DidierKabir, MorvaridToyoda, MasashiKablak-Ziembicka, AnnaTraina, GiovannaKaczyńska, KatarzynaTramice, AnnabellaKafarski, PawelTramonti, AngelaKafarski, PawełTramutola, AntonellaKagan, Isabelle A.Trebusak Podkrajsek, Kata-rinaKaji, NoriyukiTrefz, PhillipKalaitzakis, EmmanouilTrentini, AlessandroKalas, WojciechTretter, LaszloKalb, ReinhardTreviño, Claudia L.Kalemba-Drożdż, Małgor-zataTrevisson, EvaKalinova, JanaTrewin, AdamKalinowska, MonikaTribulova, NarcisKaltsa, OlgaTripathi, AshutoshKaltsatou, AntoniaTrombetta, Maria FedericaKam, AntonyTroncoso, A.M.Kamiloglu, SenemTroppmair, JakobKampf, GünterTrybek, GrzegorzKanakis, CharalambosTrzcińska, MonikaKandasamy, PalanivelTsai, Chen-ChiKanellos, PanagiotisTsai, Kun-LinKang, Jae SeungTsai, Po-JungKang, Ju-SeopTsakmakidis, Ioannis A.Kang, KyuhoTsalouchos, ArisKang, Kyung PyoTsantarliotou, Maria PKang, Min-JungTse, William K.F.Kantorow, MarcTseng, Chih-HuaKaplan, PeterTseng, Ping-HuiKaplan, ShaunaTsikouras, PanagiotisKapravelou, GaryfalliaTsingotjidou, AnastasiaKapusta, IreneuszTsironi, FannyKarbownik, Michal S.Tsironi, TheofaniaKardassis, DimitrisTsoukalas, DimitrisKardos, JuliannaTsunoda, MakotoKarmali, AminTu, Hung-PinKarousou, EugeniaTundis, RosaKarwowska, MałgorzataTuñón, María JKarwowski, BoleslawTura-Ceide, OlgaKasamatsu, ShingoTurillazzi, EmanuelaKasapidou, EleniTurkalj, MirjanaKaschina, ElenaTurkiewicz, Igor PiotrKase, SatoruTurmukhametova, NinaKashanchi, FatahTurrini, EleonoraKashfi, KhosrowTurturice, Benjamin ArthurKashino, GenrouTuttolomondo, AntoninoKasimanickam, Rama-nathanTvrda, EvaKasperkiewicz, KatarzynaTvrdá, EvaKasprzak, KamilaTyszka-Czochara, Mal-gorzataKasprzak, Magdalena PaulinaTzanavaras, Paraskevas D.Kass, MarkoTzang, Bor-ShowKassayová, MonikaTzeng, Shiang-JongKataoka, HiroyukiUchimura, KoheiKato, FumihiroUehara, MarikoKatranidis, AlexandrosUllevig, SarahKatsoris, PanagiotisUl'yanovskiy, NikolayKavanagh, DeanUngurianu, AncaKawakami, AkinoriUsai, DonatellaKawakami, SatoshiUshio-Fukai, MasukoKawamukai, MakotoUsuda, HarukiKawamura, AkiraUusi-Oukari, MikkoKawanami, DaijiVabulas, R. MartinKayano, Shin-ichiVacca, RosaKayser, KlausVaclavikova, RadkaKazdova, LudmilaVafiadaki, ElizabethKazimierczal, RenataValacchi, GiuseppeKe, Liang-YinValachová, KatarínaKe, Po-YuanValachovic, MartinKędziora, AnnaValachovičová, MartinaKeller, AdrianValasi, IreneKelterer, Anne-MarieValenti, MariaKempf, KerstinValentine, Rudy J.Kendal-Wright, Claire E.Valera-Gran, DesiréeKennedy, RoyValerio, AlessandraKernbach, JuliusValles, Soraya L.Kerr, Brian JayVallesi, AdrianaKesari, Kavindra KumarVamanu, EmanuelKęska, PaulinaVan Emon, MeganKeum, Young SamVan Setten, Gysbert BothoKeutgen, NorbertVanacker, HélèneKhalil, AbdelouahedVannini, AndreaKhambu, BilonVarea, EmilioKhan, AmjadVarela-Ramirez, ArmandoKhanam, ArshiVargas-Bello-Pérez, EinarKhasabov, Sergey G.Varghese, MathewsKhoury, DaliaVarricchio, EttoreKi, Sung HwanVarvaresou, AthanasiaKiang, Juliann G.Vasin, Mikhail V.Kicińska, AnnaVassalle, CristinaKiec-Kononowicz, KatarzynaVaudry, DavidKieliszek, MarekVaughan, RogerKikusato, MotoiVaz, Josiana A.Kilbane, JohnVazquez Espinosa, Mer-cedesKim, Dong JoonVecchione, CarmineKim, DonginVecka, MarekKim, Don-KyuVega Alvarez, Jose AntonioKim, Hahn YoungVega, Jose MariaKim, Hong-DuckVekaria, HemendraKim, Hyung-JunVelazquez, RamonKim, In-JungVelena, AstridaKim, Ji YeonVenditti, MassimoKim, JinhaeVenditti, PaolaKim, Jong-EunVeranic, PeterKim, KaVerbanac, DonatellaKim, KijinVergara, DanieleKim, Se-JaeVerhoeven, AdrieKim, SeonghunVersino, ElisabettaKim, Seung-HyunVervandier-Fasseur, DominiqueKim, Seung-JinViapiana, AgnieszkaKim, Sung EunVicario, MartaKIM, Sung JoonVicario, NunzioKim, WookiVicente, LauraKimura, NobuyukiVider, JelenaKinney, Gregory LoydVidovic, SinisaKino, KatsuhitoVieira, Helena L.A.Kirby, ChrisVignard, JulienKirilov, PlamenVignini, AriannaKirschvink, NathalieVignolini, PamelaKishikawa, NaoyaVijayakumar, SarathKiss, AlexiVillalon, MarinaKiss, BélaVillalva, MarisolKitada, MunehiroVillaverde, GonzaloKlarskov, KlausVinceti, MarcoKleeff, JörgVincze, EvaKlem, KarelViola, ManuelaKlementieva, OxanaViolet, Pierre ChristianKlepacka, JoannaVirag, LaszloKlettner, AlexaVirgili, FabioKleuser, BurkhardVishnyakov, Innokentii E.Klewicka, ElzbietaVitale, Rosa MariaKlika, Karel D.Vitali Čepo, DubravkaKlimowicz, AdamVitturi, DarioKlinke, AnnaViuda-Martos, ManuelKljusurić, Jasenka GajdošVivarelli, FabioKloc, MalgorzataVizireanu, CameliaKlochkov, S. G.Vlamis-Gardikas, AlexiosKlotz, LarsVochita, GabrielaKluczyk, AlicjaVoon, Dominic Chih ChengKmieć, KatarzynaVoulgarelis, MichaelKnap, BojanVoulgaridou, Georgia Per-sephoniKnap, NarcyzVoziyan, PaulKnecht, Kathryn T.Vu, Danh C.Knez, DamijanVukojevic, KatarinaKo, Jiunn- LiangWagner, AnikaKobayashi, HatasuWagner, Karl-HeinzKobayashi, KazuoWagner, Kay-DietrichKobayashi, SatoruWagner, RichardKobeissy, FirasWaldron, Richard T.Koch, WojciechWallert, MariaKocsy, GáborWang, Chih-HaoKoehl, Gudrun E.Wang, David Q.-H.Koike, AtsushiWang, HaoKoike, HarukiWang, Horng-darKoike, ShinWang, HsiuyingKojima, HajimeWang, Shin-WeiKokoska, LadislavWang, Shu-HueiKokoszyński, DariuszWard, Roberta JKoksharova, Olga A.Warfel, Jaycob D.Kokubo, HirokiWarmiński, KazimierzKolasa-Wołosiuk, Ag-nieszkaWarzecha, ZygmuntKoliakos, GeorgeWebb, R. ClintonKolodziejczyk, JoannaWeber, Jonasz JeremiaszKołodziejczyk-Czepas, Jo-annaWehr, BernhardKolodziejski, Pawel AntoniWei, Ming-ChiKolomytseva, MarinaWeng, Ching FengKolovos, PetrosWeng, Hao-JuiKomatsu, SatoshiWerneburg, SebastianKondera, ElżbietaWertel, IwonaKonieczka, PawełWesołowski, MarekKonno, AyumuWest, James D.Konopka, IwonaWestenbrink, B. DaanKontek, BogdanWesterblad, HåkanKontogiorgis, ChristosWetzker, ReinhardKontonasaki, EleanaWhite, RogerKopach, OlgaWianowska, DorotaKopeć, AnetaWidemann, EmilieKopitzky, RodionWieczfińska, JoannaKopjar, MirelaWiemann, MartinKorać, PetraWilde, JerzyKordek, AgnieszkaWilkins, HeatherKorus, JarosławWilkinson, DerekKorybalska, KatarzynaWilliams, Carey A.Kosmala, MonikaWilliamson, GaryKosová, KláraWilson, Jamie L.Kostara, Christina E.Wilson, Robin A.Kostov, GeorgiWindshügel, BjörnKostrzewa-Nowak, DorotaWiniarczyk, MateuszKostyn, KamilWiniarska-Mieczan, AnnaKosuge, YasuhiroWinn, NathanKotake-Nara, EiichiWińska, KatarzynaKotani, KazuhikoWirkowska-Wojdyła, MagdalenaKotlyarova, AnastasiiaWitkowska-Pilaszewicz, OlgaKotnik, PetraWitmans, ManishaKotynia, AleksandraWłodarczyk, MaciejKoulen, PeterWłodarczyk, MartaKounis, Nicholas GWłodarczyk-Stasiak, Mar-zenaKoutelidakis, Antonios E.Włodarek, DariuszKoval, AlexeyWnuk, MaciejKowalczyk, AdamWojaczyńska, ElżbietaKowalczyk, PawełWójciak, MagdalenaKozarski, MajaWojdyło, AnetaKoziak, KatarzynaWojewoda, MartaKozielewicz, PawelWojnicki, MarekKoziorowska-Gilun, Mag-dalenaWojtunik-Kulesza, Karoli-naKozlov, SergeyWojtunik-Kulesza, Karoli-na AnnaKozłowska, AnnaWon, Kyung JongKozłowska, JoannaWoźniak, GabrielaKrasnikov, BorisWozniak, MichalKravanja, GregorWronkowska, MałgorzataKrawczynski, KamilWrześniok, DorotaKrebs, BerntWu, Alexander THKreft, IvanWu, Chi-ReiKreiner, GrzegorzWu, Chun-ChiKristo, AleksandraWu, ChungChunKritsiligkou, ParaskeviWu, Chung-HsinKrizbai, István A.Wu, Jin-YiKrofta, KarelWu, MinghuaKrokidis, MariosWu, Ming-JiuanKropp, MartinaWu, Shan-YingKruglov, Alexey G.Wu, Shi-BeiKrulová, MagdalénaWujec, MonikaKrupa, KristenWyszkowski, MirosławKrupa, RenataXia, ChangleiKrystal, GeraldXu, JianqiangKu, Seung-YupXu, KuiKuan, Yu-HsiangYahia, MassinissaKuban-Jankowska, AlicjaYamada, KenjiKubatka, PeterYamakuchi, MunekazuKubica, PawełYamane, HisayoKubicka-Trzska, AgnieszkaYamauchi, AkiraKubicova, LenkaYang, Chien-ChungKucharczyk, DariuszYang, Chih-JenKucinska, MalgorzataYang, Chuen-MaoKuczyńska, BeataYang, Hung-ChiKuhn, ElisabettaYang, NanKujawska, MałgorzataYaremenko, Ivan A.Kujawski, RadosławYen, Feng-LinKukina, T. P.Yen, Frances T.Kumamoto, EiichiYen, Gow-ChinKumar, AmitYeste, MarcKumar, GauravYin, LupingKumita, JanetYiu, Kai-HangKunsági-Máté, SándorYochum, Gregory S.Kuo, Chia-HungYoon, Kyung ChulKuo, Sung-HsinYoshida, NoriakiKurasaki, MasaakiYoshihara, EijiKurdowska, Anna K.Yoshino, HironoriKurganov, BorisYotova, Iveta Y.Kurihara, HideyukiYuan, Min-HaoKursvietiene, LolitaYurchenko, EkaterinaKurzawa, MarzannaYzydorczyk, CatherineKus, PiotrZabetakis, IoannisKushima, MikiZabłocka-Słowińska, KatarzynaKushkevych, IvanZabot, Giovani L.Kvetnoy, Igor MoiseyevichZachut, MayaKwiatek, AgnieszkaZaffagnini, MirkoKwiecień, MałgorzataZagalaz Sánchez, María LuisaKwon, OranZakłos-Szyda, MalgorzataLa Mendola, DiegoZakrocka, IzabelaLa Rocca, Renato V.Zamfir, Carmen Lăcrămi-oaraLa Rocca, RobertoZamocky, MarcelLa Russa, DanieleZanfirescu, AncaLabazi, HichamŻarczyńska, KatarzynaLaboisse, Christian L.Zaremba-Czogalla, Mag-dalenaLabudda, MateuszZarrelli, ArmandoLăcătușu, Cristina-MihaelaZawada, KatarzynaLacina, LukasŻbikowska, AnnaLacroix, ChristopheZelina, PavolLagalwar, SaritaŽelježić, DavorLagarde, MichelZeman, MichalLai, Ping-ShanZembrón-Łacny, Ag-nieszkaLamari, Fotini N.Zempekakis, PantelisLammi, CarmenZeppa, GiuseppeLan, WenjianZerva, AnastasiaLancelin, Jean-marcZhao, DanLandi, NicolaZhou, PingLänger, ReinhardZielińska, DorotaLanning, NathanZielinska, MagdalenaLanzanova, ChiaraZigon, PolonaLapointe, JérômeZimmer, SebastianŁapok, ŁukaszZingg, Jean-MarcLarre, IsabelZito, FrancescaLaskowska, EwaZoidis, EvangelosLaskowska, MarzenaZovko Končić, MarijanaLasoń, WładysławZugaza, José LuisLászló Orbán, CsambalikZujko, Małgorzata E.Lattorff, H. Michael G.Zukiene, RasaLauberts, MarisZwolak, IwonaLauridsen, Jørgen TrankjærZych, MariaLaval-Gilly, PhilippeZylinska, LudmilaLavandera, Jose LuisZyśk, MarlenaLavin, Martin F.Żyżelewicz, Dorota


